# News and Miscellany

**Published:** 1884-10

**Authors:** 


### NEWS AND MISCELLANY.

### THE ACT ESTABLISHING A BUREAU OF ANIMAL INDUSTRY.

SECTION 1. *Be it enacted by the Senate and House of Representatives of the United States of America in Congress assembled,* That the Commissioner of Agriculture shall organize in his department a Bureau of Animal Industry, and shall appoint a chief thereof, who shall be a competent veterinary surgeon, and whose duty it shall be to investigate and report upon the condition of the domestic animals of the United States, their protection and use, and also inquire into and report the causes of contagious, infectious, and communicable diseases among them, and the means for the prevention and cure of the same, and to collect such information on these subjects as shall be valuable to the agricultural and commercial interests of the country; and the Commissioner of Agriculture is hereby authorized to employ a force sufficient for this purpose, not to exceed twenty persons at any one time. The salary of the chief of said Bureau shall be $3,000 per annum; and the Commissioner shall appoint a clerk for said Bureau, with a salary of $1,500 per annum.

SEC. 2. That the Commissioner of Agriculture is authorized to appoint two competent agents, who shall be competent stock raisers or experienced business men, familiar with questions pertaining to commercial transactions in live stock, whose duty it shall be, under the instructions of the Commissioner of Agriculture, to examine and report upon the best methods of treating, transporting, and caring for animals, and the means to be adopted for the suppression and extirpation of contagious pleuro-pneumonia, and to provide against the spread of other dangerous, contagious, infectious, and communicable diseases. The compensation of said agents shall be at the rate of $10 per diem, with all necessary expenses while engaged in the actual performance of their duties under this act, when absent from their usual place of business or residence as such agent.

SEC. 3. That it shall be the duty of the Commissioner of Agriculture to prepare such rules and regulations as he may deem necessary for the speedy and effectual suppression and extirpation of said diseases, and to certify such rules and regulations to the executive authority of each State and Territory, and invite said authorities to coöperate in the execution and enforcement of this act. Whenever the plans and methods of the Commissioner of Agriculture shall be accepted by any State or Territory in which pleuro-pneumonia or other contagious, infectious, or communicable disease is declared to exist, or such State or Territory shall have adopted plans and methods for the suppression and extirpation of said diseases, and such plans and methods shall be accepted by the Commissioner of Agriculture, and whenever the Governor of a State or other properly constituted authorities signify their readiness to coöperate for the extinction of any contagious, infectious, or communicable disease, in conformity with the provisions of this act, the Commissioner of Agriculture is hereby authorized to expend so much of the money appropriated by this act as may be necessary in such investigations, and in such disinfection and quarantine measures as may be necessary to prevent the spread of the disease from one State or Territory into another.

SEC. 4. That in order to promote the exportation of live stock from the United States, the Commissioner of Agriculture shall make special investigation as to the existence of pleuro-pneumonia, or any contagious, infectious, or communicable disease, along the dividing line between the United States and foreign countries, and along the lines of transportation from all parts of the United States to ports from which live stock are exported, and make report of the results of such investigation to the Secretary of the Treasury, who shall, from time to time, establish such regulations concerning the exportation and transportation of live stock as the results of said investigations may require.

SEC. 5. That to prevent the exportation from any port of the United States to any port in a foreign country of live stock affected with any contagious, infectious, or communicable disease, and especially pleuro-pneumonia, the Secretary of the Treasury be and is hereby authorized to take such steps and adopt such measures not inconsistent with the provisions of this act, as he may deem necessary.

SEC. 6. That no railroad company within the United States, or the owners or masters of any steam or sailing, or other vessel or boat, shall receive for transportation, or transport from one State or Territory to another, or from any State into the

District of Columbia, or from the District into any State, any live stock affected with any contagious, infectious, or communicable disease, and especially the disease known as pleuro-pneumonia; nor shall any person, company, or corporation deliver for such transportation to any railroad company or master or owner of any boat or vessel, any live stock, knowing them to be affected with any contagious, infectious, or communicable disease; nor shall any person, company, or corporation drive on foot or transport in private conveyance from one State or Territory to another, or from any State into the District of Columbia, or from the District into any State, any live stock, knowing them to be affected with any contagious, infectious, or communicable disease, and especially the disease known as pleuro-pneumonia; provided, that the so-called splenetic or Texas fever shall not be considered a contagious, infectious, or communicable disease within the meaning of sections four, five, six, and seven of this act, as to cattle being transported by rail to market for slaughter when the same are unloaded only to be fed and watered in lots on the way thereto.

Sec. 7. That it shall be the duty of the Commissioner of Agriculture to notify in writing the proper officials or agents of any railroad, steamboat, or other transportation company doing business in or through any infected locality, and by publication in such newspapers as he may select, of the existence of said contagion; and any person or persons operating any such railroad, or master or owner of any boat or vessel, or owner or custodian of or person having control over such cattle or other live stock within such infected district, who shall knowingly violate the provisions of section six of this act, shall be guilty of a misdemeanor, and upon conviction, shall be punished by a fine of not less than $100 nor more than $5,000, or by imprisonment for not more than one year, or by both such fine and imprisonment.

Sec. 8. That whenever any contagious, infectious, or communicable disease affecting domestic animals, and especially the disease known as pleuro-pneumonia, shall be brought into or shall break out in the District of Columbia, it shall be the duty of the Commissioners of said District to take measures to suppress the same promptly and to prevent the same from spreading; and for this purpose the said Commissioners are hereby empowered to order and require that any premises, farm, or farms where such disease exists, or has existed, be put in quarantine; to order all or any animals coming into the District to be detained at any place or places for the purpose of inspection and examination; to prescribe regulations for and to require the destruction of animals affected with contagious, infectious, or communicable disease, and for the proper disposition of their hides and carcasses; to prescribe regulations for disinfection, and such other regulations as they may deem necessary to prevent infection or contagion being communicated, and shall report to the Commissioner of Agriculture whatever they may do in pursuance of the provisions of this section.

SEC. 9. That it shall be the duty of the several United States District Attorneys to prosecute all violations of this act which shall be brought to their notice or knowledge by any person making the complaint under oath; and the same shall be heard before any district or circuit court of the United States or Territorial court holden within the district in which the violation of this act has been committed.

SEC. 10. That the sum of $150,000, to be immediately available, or so much thereof as may be necessary, is hereby appropriated, out of any moneys in the treasury not otherwise appropriated, to carry into effect the provisions of this act.

SEC. 11. That the Commissioner of Agriculture shall report annually to Congress, at the commencement of each session, a list of the names of all persons employed, an itemized statement of all expenditures under this act, and full particulars of the means adopted and carried into effect for the suppression of contagious, infectious, or communicable diseases among domestic animals.

### SOME RECENT INVESTIGATIONS ON GERMS.*

(The following letter has been received at this office. The author, Herr Riechschwamm,† does not belong to our staff of special correspondents, nor is he personally known to us. He signs himself, "First Assistant to Dr. Hahn," but upon reference to the last volume of the *Mittheilungen aus dem Kaiserli-*

* The Medical News.

† "Riechschwamm," as used here, means protective or smelling sponge over the nose and mouth.

*chen Gesundheitsamte*, Magdeburg, we do not find his name among the Hülfsarbeiter. These facts, together with the very remarkable results of the investigations he reports, have led us thus to inquire into his trustworthiness. We have felt it incumbent upon us in publishing his letter to make this prefatory statement, rather than to withhold information of these latest researches, which are certainly calculated to arrest our attention even if they do not immediately commend themselves to our unreserved acceptance.—Ed.)

Magdeburg, January 31, 1884.

*To the Editor of The Medical News.*

Sir—I have thought that some account of the recent discoveries in regard to bacilli might interest readers who are remote from the great radiating centres of constructive science. The latest of these discoveries is even yet spoken of here with caution, but by the time you have printed this it will have revolutionized one branch of physiology, and produced an evolution of novel ideas, the final results of which not the boldest can predict. But before outlining for your readers a discovery which I am privileged to communicate by permission of Professor Coccischlächter,‡ and before it has been fully published at home, it may be well to describe the Krankheitenursprungsanstalts—Museum of Bacilli—collected by the Herr Oberprofessor Keimerzeurger‖ von Verdammtnarrburg.

This has been done at a cost of more than one life upon the altar of science. The museum is a room about thirty feet square, with double walls of glass, between which circulates water kept at a temperature of 30° C., by three gigantic thermostats, which are so accurate that the heat does not vary the one-fifteenth of a degree. Ranged along the sides, exposed to air or under glasses, are hundreds of half potatoes on which grow bacilli; of late, however, boiled cabbage is said by Keimerzeurger to answer better. Certain specific cocci flourish on the Beta altissima, or mangel wurzel, but as to this choice of cultur gartens more is to be said. To walk through this museum with the Herr Professor Keimerzeurger is interesting.

Before entering, a mask is given you, and a bottle of condensed oxygen, so as to enable you not to inhale the atmos-

‡ "Coccischlachter." Death to Cocci.
‖ "Keimerzeurger." Germ-generator.

phere loaded with germs. In tones muffled by the need to speak within the mouth-piece, you learn that to the left is a tubercular potato, its surface gray with the potencies of countless deaths. Near it the bacillus of gonorrhœa flourishes on the cut surface of the succulent beet, beside the ruddy germs of syphilis. Scarlet fever infests this potato, diphtheria that. The new bacteria of pneumonia flourishes on a boiled watermelon; and glanders, cholera, smallpox, and hydrophobia spread in tiny greenish growths over the little gardens of gelatine.

For a moment, in my interest, I displaced my mask. The professor instantly seized me and hurried me from the room. "What a risk!" he said; "my last assistant did as you did and he died in seven days of acute phthisis, with symptoms of hydrophobia and whooping cough, combined in horrible equality." I did not desire to reënter this box of Pandora. In adjoining apartments of less size are the experimental cultivations, those which are still in doubt. Among the most interesting is the micrococcus of gout, found to flourish best upon gelatinized turtle soup.

A most striking practical result has grown out of some of Coccischlächter's and Keimerzeurger's later researches. They have been able to show that the bacterium of colic flourishes on the green apple, which accounts for the gripes experienced by youthful gourmands. But far more remarkable is the fact, that certain micrococci and bacteria die, as proved by Coccischlächter, in some culture materials, and thrive, as shown by Keimerzeurger, on others. Thus the tubercle bacilli flourish on boiled cabbage (Keimerzeurger), but perish on moist sauerkraut (Coccischlächter), so that by a persistent diet of the latter article they have been able to saturate some of their devoted assistants up to the point of insusceptibility, a discovery which will, we trust, put an end to the cavils at the failure of these researches to yield practical results.

Most startling of all is Herr Keimerzeurger's latest result. He has been able to show that the inconvenient monthly sickness known as the menstrual flow is a non-essential of female life, and is due to a peculiar bacillary growth. It is at once and for many months destroyed by the growth of the bacterium virilis, long misapprehended as the spermatozooid. This latest discovery has been received with derision in France, but Hahn has given it his support, and in my next I shall report his confirmatory experiments.

RIECHSCHWAMM,
First Assistant to Dr. Hahn.

